# Integrating 3-D thermal videography, ultrasonic acoustics, and weather radar to characterize bird and bat activity at wind turbines

**DOI:** 10.1371/journal.pone.0352329

**Published:** 2026-07-14

**Authors:** Abigail Shultz, Donald Solick, Michael Whitby, Christian Newman, Aaron Corcoran

**Affiliations:** 1 Department of Biology, University of Colorado Colorado Springs, Colorado Springs, United States of America; 2 Electric Power Research Institute, Palo Alto, California, United States of America; 3 Bat Conservation International, Austin, Texas, United States of America; San Diego Zoo Institute for Conservation Research, UNITED STATES OF AMERICA

## Abstract

Wind turbines intersect airspace used by both migratory birds and bats, yet most monitoring approaches rely on single sensing modalities that capture only part of this system. Here, we integrate synchronized three-dimensional (3-D) thermal videography, ultrasonic acoustic monitoring, and regional weather radar data to characterize wildlife activity at two inland wind turbines during fall migration in Iowa, USA. Across 38 nights in late summer and autumn of 2022, we recorded 12,047 3-D flight tracks within rotor-swept altitudes, 2,249 bat echolocation sequences, and cumulative radar-derived migration traffic of approximately 2.0 million birds/km. Thermal video detections were strongly correlated with radar-derived migration intensity but not with acoustic detections. This pattern is consistent with birds comprising the majority of thermal video detections during peak migration. Thousands of flight trajectories occurred within 30–150 m above ground level and within 200 m of turbine monopoles, demonstrating frequent use of altitudes associated with collision risk. Fine-scale trajectory analysis revealed strong avoidance of flight paths directed toward the rotor-swept zone (RSZ), with targets 3.6 times more likely to fly toward the RSZ when turbines were stationary than when producing power. Angular divergence from turbine bearing also increased with decreasing distance, with a steeper avoidance gradient near operating turbines, consistent with birds actively responding to cues associated with blade rotation. Our results demonstrate how a sensor-fusion framework improves inference about taxonomic composition, airspace use, and behavioral responses near wind turbines. The strong relationship between regional radar activity and turbine-level detections highlights the potential of publicly available radar data to support wind energy siting by identifying areas with lower migratory bird traffic without extensive on-site monitoring. Understanding the sensory basis of the avoidance behavior documented here could also inform the design of deterrent systems for infrastructure where bird collisions are a concern. Integrating complementary monitoring technologies provides a scalable approach for understanding wildlife–turbine interactions and guiding responsible wind energy development.

## Introduction

Wind energy development is expanding rapidly across North America and globally, increasing the spatial overlap between wind turbines and the airspace used by volant wildlife. Both birds and bats experience mortality at wind turbines, although the mechanisms driving risk differ across taxa and environmental conditions [1–3]. Quantifying how these animals use turbine airspace—particularly at night, when both bird migration and bat activity peak—remains a central challenge for understanding and mitigating collision risk.

Most assessments of wildlife–turbine interactions rely on carcass searches, which provide estimates of mortality but limited insight into the behavioral processes that precede collisions [4–7]. Alternative monitoring technologies offer complementary perspectives but also have inherent limitations. Weather radar networks, such as the U.S. NEXRAD system [8], provide broad-scale estimates of migration intensity and direction across regions, but lack the spatial resolution necessary to resolve fine-scale interactions with individual turbines [9–14]. Ultrasonic acoustic detectors offer taxonomically specific information about bat activity but do not detect birds and provide limited spatial context [15–20]. Additionally, there is the potential complication of acoustic undersampling of species and accurately converting call counts to the number of individuals [21,22]. Thermal imaging can directly visualize birds and bats flying within the rotor-swept zone (RSZ) [16,23–25], and stereoscopic systems allow reconstruction of three-dimensional flight trajectories [26–29], yet thermal detections alone often cannot reliably distinguish birds from bats without corroborating information [14,29].

Because each sensing modality captures different aspects of animal movement, integrating multiple systems may provide a more complete understanding of how birds and bats use turbine airspace [30–32]. In particular, comparing temporal and directional patterns across radar, acoustics, and high-resolution 3-D thermal video offers an opportunity to (1) determine which taxa dominate detections within rotor-swept altitudes at different times of season, (2) evaluate concordance between regional migration intensity and local turbine-level activity, and (3) quantify fine-scale behavioral responses to turbines that are invisible to coarser monitoring approaches.

Inland wind farms are of particular interest because they intersect major migratory flyways used by billions of nocturnally migrating birds and bats each year [1,3,33–35]. While offshore studies and inland diurnal migrants have documented large-scale avoidance of wind farms by some species [36–42], comparatively less is known about how nocturnal migrants respond to individual inland turbines. Birds migrating at night often travel at high altitudes, but atmospheric conditions can compress flight altitudes, potentially placing migrants within the RSZ [6,43]. At the same time, migratory bats are known to frequent turbines, particularly during late summer and early autumn, yet their temporal patterns and spatial behaviors may differ from those of birds [1–3,44]. Some studies suggest that due to habitat disturbance from turbine development, there is a reduction in nocturnal bat activity in nearby hedgerows [45–48], though behavior at turbines is more variable and less understood. Understanding how these taxa partition time and airspace near turbines is critical for interpreting monitoring data and assessing risk.

High-resolution 3-D flight trajectories offer a means of examining not only whether animals occur within rotor-swept altitudes, but how they behave as they approach turbines [25,27,49]. Fine-scale deviations in flight direction may reveal avoidance responses that reduce collision probability, whereas persistent approach trajectories could indicate heightened risk. However, without broader temporal and regional context, such local observations are difficult to interpret. Sensor fusion—linking turbine-level trajectory data with acoustic monitoring and regional radar indices—provides a framework for resolving these uncertainties.

Here, we integrate synchronized 3-D thermal videography, ultrasonic acoustic monitoring, and regional weather radar data to characterize bird and bat activity at two inland wind turbines during fall migration. Specifically, we (1) compare temporal patterns of activity across sensors to assess concordance and seasonal partitioning between birds and bats, (2) evaluate the relationship between regional radar-derived migration intensity and turbine-level detections, and (3) quantify flight trajectories of targets approaching turbines to assess whether behavioral avoidance of the rotor-swept zone differs when turbines are producing power versus stationary. By explicitly comparing these complementary sensing modalities, this study provides a mechanistic perspective on how volant wildlife use turbine airspace and demonstrates how integrated monitoring approaches can improve inference about risk at wind energy facilities.

## Materials and methods

### Data collection

**Fig 1:**
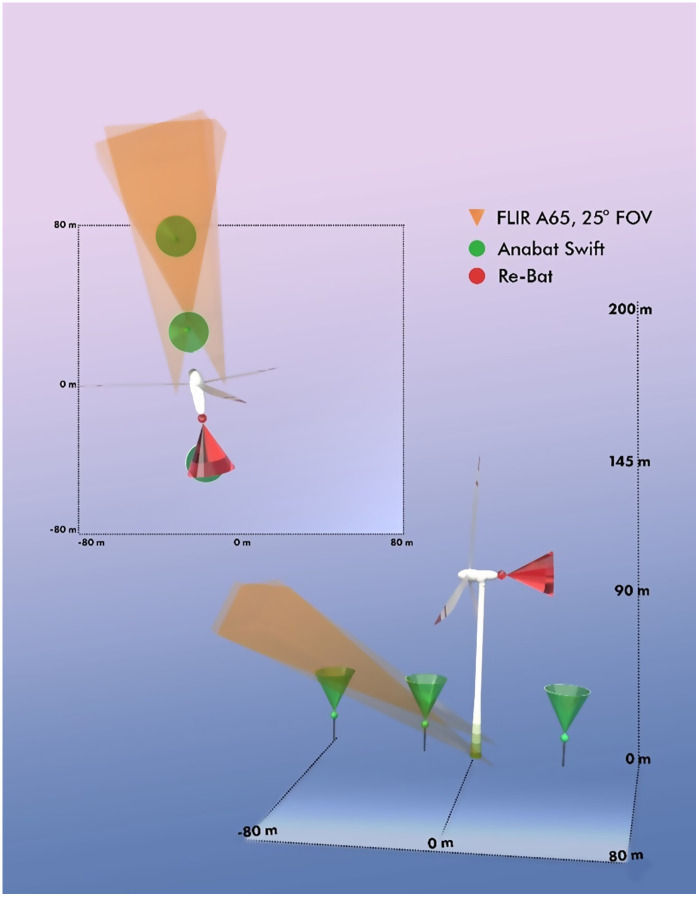
Visualization of locations and spatial sampling and thermal video (orange) and ultrasonic acoustic sensors deployed at wind turbines. Overhead (top) and 3-D perspective (bottom) views are shown. Cones of detection for Anabat and Re-Bat microphones are approximate and are not meant to reflect the precise detection ranges or volumes.

We collected ultrasonic acoustic and thermal video data at two Vestas V110 2.0 MW turbines at Orient Wind Farm in Adair County, Iowa from August 20th to October 7th, 2022. The wind turbines had a hub height of 95 m and rotor diameter of 110 m, placing the RSZ 40–150 m above ground level (Fig 1). At each turbine, we placed two Flir A65 thermal cameras (640 x 512-pixel resolution and 25° field of view) synchronized electronically with an M12 synch cable, recording the airspace adjacent to the turbines nightly from sunset to sunrise. Because each camera’s narrow field of view captures only a limited slice of the surrounding airspace, we oriented both cameras to view the north-northwest of the turbines (330°), based on prevailing southerly winds during fall migration and the expectation that nocturnally migrating birds and bats would predominantly approach turbines from the north. The cameras were 40 m apart and angled slightly inward to provide overlapping fields of view starting approximately 60 m away from the cameras. We aimed the cameras 30° up from the horizon to capture an ascending slice of airspace adjacent to the wind turbine and document bats and birds that could be approaching the RSZ and at risk of collision.

At each turbine, we deployed three Anabat Swift ultrasonic detectors (Titley Electronics, Columbia, Missouri) on 7 m poles mounted in the ground. We placed two detectors 30 m and 70 m north-northwest of the monopole and the final detector 30 m south-southeast of the monopole to document bat activity along an axis corresponding to the aim of our cameras. Two ReBAT ultrasonic detectors were mounted to the end of each turbine nacelle. One microphone was angled below the nacelle and one angled above the nacelle. The detectors recorded calls from 30 minutes before dusk to 30 minutes after dawn. For additional information on methodology, see [50].

**Ethics statement.** All data were collected using non-invasive remote-sensing techniques (thermal videography, ultrasonic acoustic detectors, and publicly available weather radar). No animals were captured, handled, or otherwise disturbed during the study, and the work did not involve protected species or sites. Accordingly, no institutional animal care and use committee (IACUC) approval or permit was required. Site access was granted by MidAmerican Energy Company.

We obtained NEXRAD Level II radar data from the KDMX (Des Moines, Iowa) station via Amazon Web Services. The KDMX radar is located approximately 83 km northeast of the Orient Wind Farm, beyond the ~ 35 km range within which bioRad’s vertical profile retrievals are typically considered most reliable [51]. Because of this distance we used radar activity as a regional index of migration intensity integrated across the broader radar-coverage area, rather than as a site-specific measurement of activity directly above the turbines. Vertical profiles of bird migration were generated by Adriaan M. Dokter using the vol2birdR and bioRad packages [51] and provided as regularized vertical profile time series with 100 m altitude bins. We integrated profiles using the integrate_profile command with default altitude bounds (0–5 km) to derive migration traffic rate (MTR; birds/km/hour), ground flight direction, ground speed, and wind speed and direction. To evaluate whether restricting the radar to altitudes closer to the thermal video window (30–150 m above ground level) would more closely match turbine-level activity, we also re-integrated the profiles with alt_max set to 150, 300, 500, 1,000 and 2,000 m. The KDMX vertical profile product does not return density values below 300 m AGL (the 0, 100 and 200 m bins were all NA), likely as a consequence of beam-elevation geometry and ground-clutter filtering applied during VPR construction; an exact altitude match to the thermal window (30–150 m) was therefore not possible. The lowest-altitude comparison available (alt_max = 500 m, which includes the 300–500 m bins) yielded a correlation with nightly thermal detections (Pearson r ≈ 0.67) that was not improved by raising the cap further (r = 0.67–0.68 across alt_max = 500–5,000 m; S1 Text); we therefore retained the default 0–5 km integration for the main analyses. We converted timestamps from UTC to local time (America/Chicago) and filtered data to nighttime hours (sunset to sunrise) during the study period (August 20–October 7, 2022). Rows with missing values were excluded. We estimated total regional migration passage by summing MTR values across the sampling period.

### Data processing

We processed thermal video using the MATLAB [52] software package ThruTracker [26] with an updated You Only Look Once (YOLO) version 3 [53] algorithm to automatically detect objects and create 2-D tracks for each camera view using uncompressed background subtracted videos. We calibrated camera intrinsics by taking images of a heated metal plate that had a matrix of circular holes at known positions [50], and generated camera extrinsics using animal flight paths as background points [26]. Three-dimensional positions were reconstructed by triangulating matched 2-D detections across the two overlapping, frame-synchronized camera views using a direct linear transformation (DLT) calibration [54]. Altitude and three-dimensional position therefore derive from stereo parallax and camera geometry alone; target size and flight speed are not used as altitude proxies, so inter-individual variation in those traits does not bias altitude estimates. Our automated algorithms generated 3-D detections, which we manually reviewed. Calibration accuracy was assessed by the reprojection residual of reconstructed 3-D points, which was typically < 2 pixels. Given the camera resolution (640 × 512 pixels; ~ 25° × 18° field of view), this corresponds to spatial errors of approximately 0.07 m at 50 m and 0.26 m at 200 m, well within the precision required for the distance categories used in subsequent analyses.

All additional data processing was conducted using custom code written in RStudio [55] version 4.4.1 [56]. Circular statistics were calculated using the circular package [57], managing and manipulating the date-time data was completed using the lubridate package [58]. The package dplyr [59] was used for data manipulation and transformation. The tidyr package [60] was used for data cleaning and organization. Additional data visualization utilized the ggplot2 package [61].

We filtered thermal detections to include only tracks occurring within 60–200 meters of the turbines and at altitudes between 30–150 meters, as we were not confident of our ability to detect bats and birds outside of these distances. These filters also target altitudes and ranges at which insect and aircraft detections are uncommon in our thermal data (A. Corcoran, unpublished data); however, they do not fully exclude such targets, and we retain the possibility that a small number of non-target detections remain in the dataset. Thermal video detections were matched with turbine Supervisory Control and Data Acquisition (SCADA) weather data at the time of detection, which included wind speed and wind direction. Additionally, each detection was linked to a specific turbine ID and assigned three-dimensional coordinates (x, y, z) relative to the turbines.

We processed thermal video flight trajectories to extract key flight parameters, including altitude and ground direction. Ground flight direction was calculated from the start and end positions of each 3-D trajectory. We derived tailwind and crosswind components for each track using SCADA wind direction and the track’s ground direction. Tracks were classified as wind-assisted when the tailwind component (i.e., the component of wind speed in the direction of flight) was greater than zero, and not wind-assisted when the tailwind component was less than zero. To quantify the proportion of wind-assisted flights, we categorized wind conditions into four directional regions based on the direction winds were blowing toward: north (315°–45°), east (45°–135°), south (135°–225°), and west (225°–315°). To avoid using circular wind direction variables in our statistical models, we converted wind speed and direction measurements into north-south and east-west wind speed components. Thermal video data were further summarized into date–hour intervals, including the sums for thermal video counts, thermal video counts by turbine ID, and weather and detection flight parameter averages. For radar data, average crosswind and tailwind components were calculated from the 15-minute aggregated estimates following the same approach. To avoid biasing our data, we only included time periods when cameras were operational at both wind turbines. Radar was processed for each date hourly interval, including radar counts that were calculated by averaging all the 15-minute MTRs, and radar flight parameters were also averaged for each date’s hourly intervals. This resulted in 303 hours of video recorded across 38 nights within the study period.

All acoustics were processed automatically to filter out noise using Sonobat 30.0 (Arcata CA, USA). We conducted an additional round of manual review using an expert in bat acoustics with over 20 years of experience to confirm all accepted detections were bats. We aggregated acoustic detections across all ground and nacelle-mounted detectors hourly as an index of temporal bat activity patterns.

The resulting hourly summaries for thermal video, radar, and acoustics were filtered to include only data from sunset to sunrise from 20 August to 7 October, 2022. Any hourly interval that had no thermal video or radar information was excluded, and any hourly interval that had information for only one turbine was excluded.

### Statistical analysis

To assess relationships between sensor modalities, we summarized thermal video detections, acoustic detections, and radar-derived MTR as nightly totals (sunset to sunrise). We did not analyse these relationships at the hourly scale because hourly detection counts within nights were strongly temporally autocorrelated (Ljung–Box test on residuals of an hourly log–log model of thermal counts on MTR: Q₁₀ = 289, p < 10 ⁻ ¹⁶; lag-1 residual autocorrelation ≈ 0.68), violating the assumption of independent observations that underlies the Tweedie GLM framework used here. Nightly totals match the biologically relevant timescale of nocturnal migration events and showed no detectable residual autocorrelation (Ljung–Box Q₁₀ = 7.1, p = 0.71; see S2 Text). We modeled relationships using Generalized Linear Models (GLMs) with the Tweedie package and log-link function [62]. To characterize pairwise relationships among all three sensor modalities, we first fit three univariate models: (1) thermal video ~ radar MTR, (2) thermal video ~ acoustic detections, and (3) radar MTR ~ acoustic detections. We then fit a Tweedie log-linked multivariate model (thermal video ~ radar MTR + acoustic detections) to evaluate whether bat acoustic activity explained additional variation in thermal detections beyond radar-derived migration intensity. We determined the multivariate significance using deviance explained (DE) and compared it to the univariate radar MTR model by fitting it with an ANOVA test.

We examined flight behavior of targets on video and on radar by generating polar histogram plots of ground flight direction relative to wind direction when winds blew north (315°–45°), east (45°–135°), south (135°–225°), and west (225°–315°), also quantifying the proportion of flights with or against the wind under each condition.

To assess behavioral responses of targets approaching wind turbines, we filtered tracks to tailwind conditions in which winds blew targets directly toward the turbines as viewed by the cameras (cameras facing 330°; SCADA wind direction 135°–165°). For each track, we calculated the overall ground flight direction from start to end positions and the median three-dimensional position relative to the turbine.

We quantified avoidance using two complementary approaches. First, we defined a RSZ “danger zone” for each track based on the angular extent of the RSZ as seen from the target’s median position. Tracks whose ground flight direction fell within the angular bounds of the RSZ were classified as RSZ-directed. We classified turbine operational status from SCADA data as either rotating (producing power) or stationary (not producing power) and compared the proportion of RSZ-directed flights between conditions using a generalized linear mixed model (GLMM; RSZ-directed ~ operational status + turbine + (1 | night), binomial family with logit link; lme4 package, [63]). Turbine identity (2 levels) was included as a fixed effect rather than a random effect for two reasons: (i) random-effect variance components require multiple grouping levels (typically ≥ 5–6) for reliable estimation, and (ii) the two turbines represent the full set of turbines monitored at this site rather than a random sample from a broader turbine population, making fixed-effect treatment inferentially appropriate. Night was included as a random intercept to account for repeated measures within nights. Stationary periods resulted from multiple causes, including wind speeds below the cut-in threshold, experimental curtailment treatments applied during the study period, and other operational constraints. This variation means that stationary conditions were not exclusively associated with low wind speeds. We report the odds ratio with 95% confidence interval as a measure of effect size.

Second, to test whether avoidance increased with proximity to turbines and differed by operational status, we modeled the absolute angular deviation of each track’s flight direction from the bearing to the turbine as a function of distance and operational status using a generalized additive model (GAM; Gaussian family, identity link) fit with the mgcv package [64]. We fit the model with separate smooth terms for distance under each operational status, a fixed effect for turbine, and a penalized random intercept for night (i.e., angular_deviation ~ s(distance, by = status) + status + turbine + s(night, bs = “re”)), allowing the distance–avoidance relationship to vary between rotating and stationary conditions. We evaluated model fit using deviance explained and compared models with and without the operational status term using AIC.

For all statistical analyses, we set alpha to 0.05.

## Results

### Comparison of radar, video and acoustic activity patterns

We recorded 12,047 3-D flight tracks of either birds or bats (hereafter “targets”) on thermal video, 2,249 bat echolocation sequences (including 1,208 detections from nacelle-mounted ReBATs and 1,041 detections from ground-mounted Swift detectors), and a cumulative radar-derived regional migration traffic of approximately 2.0 million birds/km over 38 nights of our study period. Hourly thermal video detection rates were highly correlated between our two study turbines (Pearson’s correlation coefficient = 0.92); therefore thermal video detection rates across turbines were pooled for further analysis.

**Fig 2:**
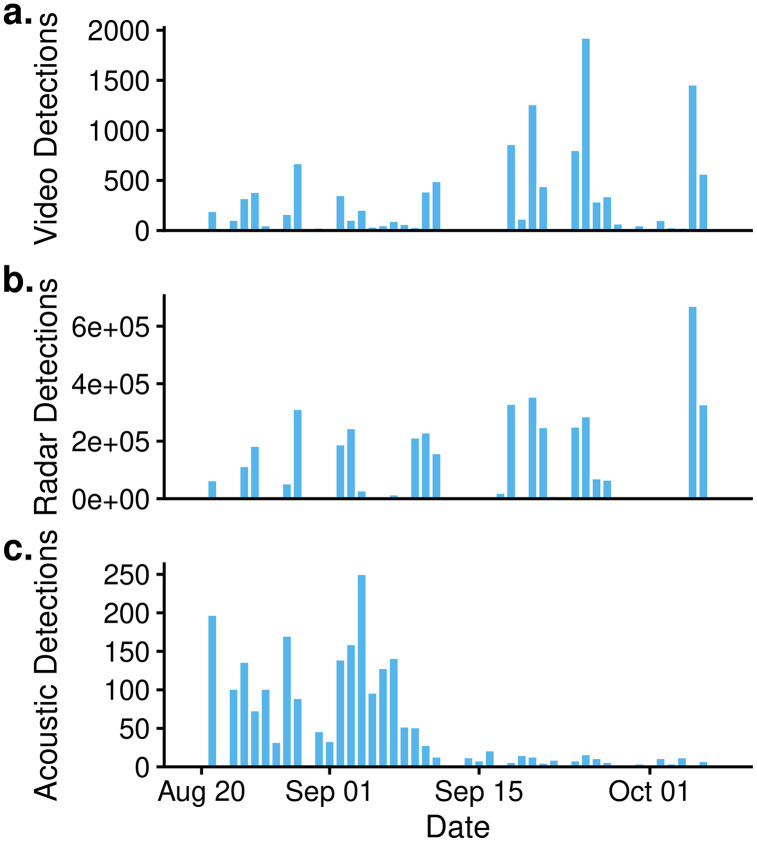
Nightly activity levels recorded by (a) thermal video, (b) radar, and (c) ultrasonic acoustics (c) across the sampling period.

**Fig 3:**
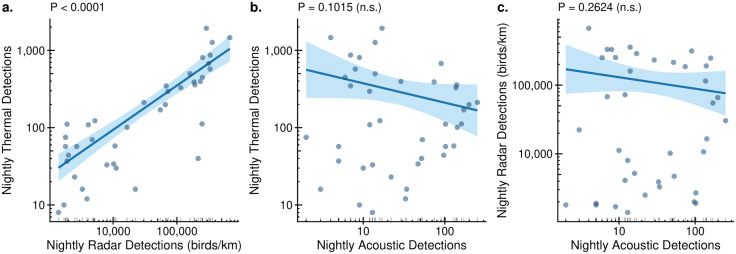
Relationships between nightly thermal video, ultrasonic acoustic and radar detections. Comparisons are shown between (a) thermal video and radar, (b) acoustics and thermal video, and (c) acoustics and radar based on Tweedie log-linked GLMs. Axes are displayed on a log scale; tick labels give values on the original (untransformed) scale. Solid lines are fitted values from the GLM and shaded bands are 95% confidence intervals on the fitted mean.

Nightly activity levels were higher in late September and October for thermal video and radar detections, whereas acoustic detections were higher in August and early September (Fig 2). High nightly variability was observed in data collected from all three sensors. We modeled pairwise nightly correlations among sensors using Tweedie GLMs with a log link, estimating the Tweedie index parameter for each model via maximum profile likelihood (all *p* = 1.82–1.95, indicating compound Poisson–gamma distributions near the gamma end of the family, consistent with continuous, right-skewed nightly totals). Radar and thermal video detection rates were highly correlated (Tweedie GLM, Tweedie index = 1.88, *φ* = 0.93; *p* < 0.001, DE = 0.72; Fig 3a) with a positive and mostly linear relationship, whereas acoustics were not correlated to thermal video (Tweedie index = 1.95, *φ* = 2.30; *p* = 0.10, DE = 0.06; Fig 3b) or radar (Tweedie index = 1.95, *φ* = 2.85; *p* = 0.26, DE = 0.02; Fig 3c). A multivariate Tweedie model (Tweedie index = 1.82, *φ* = 1.24) with both radar and acoustics as predictors of thermal video detections confirmed that radar was the dominant predictor (*p* < 0.001) while acoustics remained non-significant (*p* = 0.24), with only marginal improvement in deviance explained (DE = 0.73 vs. 0.72). All results for predicting thermal video activity from acoustic activity remained non-significant (p > 0.10) when running the same analyses with only the ground or only the nacelle-mounted acoustic detectors. These relationships are consistent with birds comprising a substantial proportion of thermal video detections, particularly late in the season during peak bird migration.

### Flight directions and wind assistance in thermal video and radar

**Fig 4:**
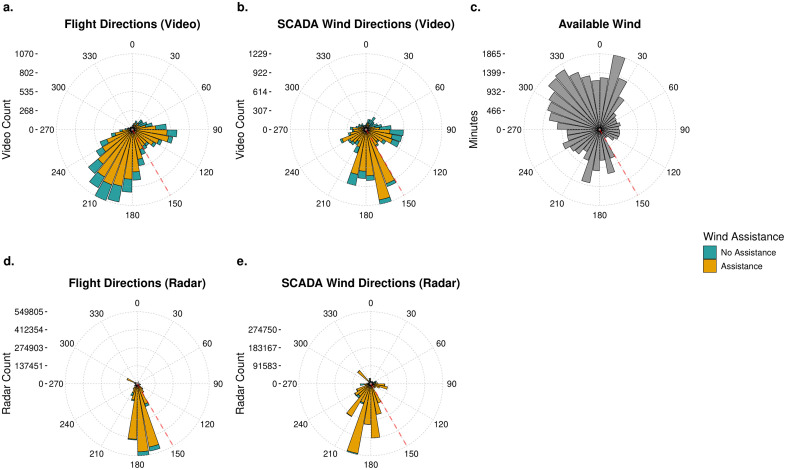
Flight and wind directions from thermal video and NEXRAD radar. Ground flight directions are shown for (a) thermal video flights and (d) NEXRAD radar; SCADA wind directions are shown during (b) thermal video flights and (d) radar events and (c) the sampling period of the study. Events are colored by presence or absence of wind support (i.e., positive vs. negative tailwind).

For all video detections, 78.7% of thermal video flights had wind support. Wind support was highest with winds blowing south (87.9% of 6,066 flights) and west (84.0% of 2,274 flights). Winds blowing east provided lower wind assistance (67.4% of 3,401 flights), whereas winds blowing north provided support for only slightly more than half of flights (53.3% of 1,181 flights; S1 Fig). For both thermal video (Fig 4b) and radar (Fig 4e), most flights occurred when winds blew south, with a secondary peak of activity when winds blew east. This was despite most wind during the study period blowing north (Fig 4c). Radar flights showed a strong unimodal peak of birds almost directly south (Fig 4d). In contrast to radar, thermal video flight trajectories showed a clear bimodal distribution, with most flights going south-southwest and fewer flying east (Fig 4a). A clear gap in activity occurred between these peaks, coinciding with the 150° azimuth of the wind turbine relative to the cameras. To examine whether the bimodal directional structure reflected fine-scale behavioral modification near turbines, we analyzed flight directions as a function of distance from the turbine under tailwind conditions.

### Behavioral avoidance and sensory cues

**Fig 5:**
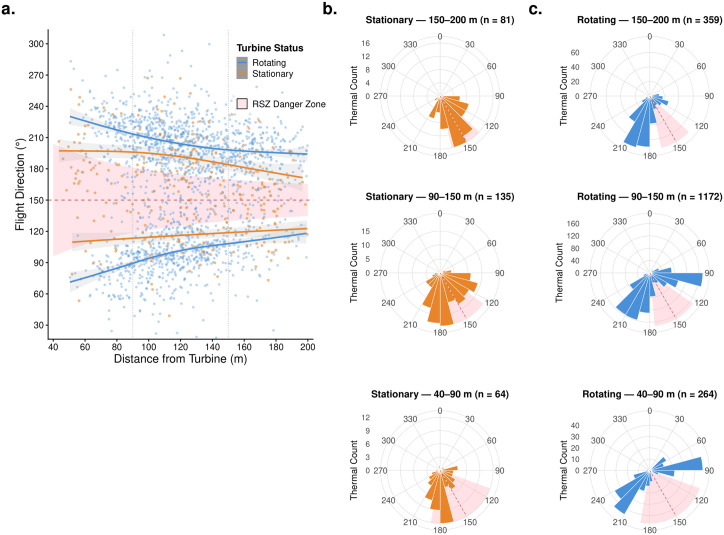
Target flight directions in thermal video flights as a function of distance from the wind turbine for flights where winds blew targets toward the wind turbines as viewed by the cameras. (a) Scatter plot of all flights coded by operational status (rotating: blue; stationary: orange). Two GAM trendlines are shown for each status, fit separately to tracks with camera-frame flight directions above (≥ 150°) and below (< 150°) the turbine bearing, reflecting the bimodal directional distribution as targets deviate to either side of the turbine. Shaded bands around each trendline are 95% confidence intervals on the fitted mean. Polar histogram plots show flight directions of targets detected at far (150–200 m), middle (90–150 m), and close (40–90 m) distances from the wind turbines for tracks where the wind turbine was (b) stationary and (c) rotating. The red shaded region indicates the flight angles at each distance that would cause a target to fly through the RSZ.

Lastly, we examined flight directions of targets when winds blew directly toward the wind turbine, as viewed by our cameras facing north-northwest (Fig 5). Flight directions showed a bimodal distribution, with most targets flying south-southwest and a secondary peak flying east, separated by a clear directional gap aligned with the bearing to the turbine (Fig 5b–c). This bimodal structure was far more pronounced when turbines were rotating and producing power than when stationary. When turbines were producing power, targets showed strong divergence from the turbine bearing (median angular deviation = 52.8°), with only 11.6% of 1,795 tracks directed toward the rotor-swept zone (RSZ). When turbines were stationary, targets flew more directly toward the turbine (median angular deviation = 32.8°), and 40.4% of 280 tracks were RSZ-directed (Fig 5a). A generalized linear mixed model (GLMM) with a random intercept for night and a fixed effect for turbine confirmed that tracks were 3.6 times more likely to be directed toward the RSZ when turbines were stationary than when rotating (OR = 3.57, 95% CI: 2.19–5.83, p < 0.001).

Angular divergence also increased with proximity to turbines under both conditions, but the gap between rotating and stationary turbines widened substantially at closer distances. At 150–200 m, the bimodal pattern was less pronounced, with median angular deviations of 43.8° (rotating) and 22.2° (stationary; Fig 5b–c, top row). From 90–150 m, targets flew progressively farther from the turbine bearing (52.1° rotating, 35.1° stationary; Fig 5b–c, middle row). At 40–90 m, the bimodal peaks were sharply defined and displaced well away from the turbine direction when turbines were rotating (68.8°), while stationary turbines elicited only modest additional divergence (36.9°; Fig 5b–c, bottom row). A GAM of absolute angular deviation with separate distance smooths by operational status, a fixed effect for turbine, and a random intercept for night (deviance explained = 17.0%, ΔAIC = −31 vs. reduced model without status) confirmed that avoidance increased with decreasing distance under both rotating (edf = 3.2, p < 2e-16) and stationary (edf = 1.1, p < 0.001) conditions, with a steeper response when turbines were producing power. In summary, targets approaching turbines with tailwinds avoided flight paths toward the RSZ, and this avoidance was substantially stronger when turbines were operating.

### Wind speed as a potential confound

Although operational status is partly linked to wind speed, stationary periods in this study also resulted from experimental curtailment and operational constraints, partially decoupling the two variables. Nonetheless, wind speeds were substantially higher during rotating conditions (median = 9.59 m/s, IQR: 8.17–10.87) than stationary conditions (median = 6.20 m/s, IQR: 4.69–7.38; Wilcoxon p < 0.001). We therefore tested whether the status–avoidance relationship could be an artifact of wind speed differences using three approaches. First, adding wind speed as a covariate to the GLMM did not attenuate the status effect (adjusted OR = 3.80, 95% CI: 2.18–6.63, p < 0.001); wind speed was a modest but significant predictor (OR = 1.17, p = 0.003), and the status × wind speed interaction was non-significant (p = 0.88). Second, adding a nonlinear wind speed smooth to the mixed GAM improved overall fit (deviance explained = 17.8%, ΔAIC = −11), but the status effect remained unchanged (coefficient = −15.1°, p < 0.001), indicating that wind speed and operational status both exert effects on flight behavior. Third, curtailment treatments created stationary periods at wind speeds well above the cut-in threshold, providing a quasi-experimental test of the blade-rotation hypothesis. Among the 171 stationary tracks recorded at wind speeds ≥ 5 m/s — conditions that would normally keep turbines spinning — 38.0% were RSZ-directed compared with 11.8% of 1,760 rotating tracks at comparable winds (OR = 4.58, 95% CI: 3.25–6.44, p < 0.001; GAM deviance explained = 13.3%, status p < 0.001). In summary, targets approaching turbines with tailwinds avoided flight paths toward the RSZ, and this avoidance was substantially stronger when turbines were operating. This result is consistent with behavioral responses to cues associated with rotating blades rather than an artifact of wind speed.

## Discussion

By integrating synchronized 3-D thermal videography, ultrasonic acoustic monitoring, and regional weather radar data, this study demonstrates how complementary sensing modalities can improve inference about wildlife use of wind turbine airspace. Each sensor independently captures only part of the system. When analyzed together, however, they provide convergent evidence about taxonomic composition, temporal dynamics, and fine-scale behavioral responses near turbines.

### Sensor concordance and taxonomic inference

This multi-sensor comparison strengthens taxonomic inference in a way that no single monitoring approach could accomplish alone [11,30,31]. Thermal imaging provides high-resolution 3-D trajectories but often cannot reliably distinguish birds from bats without corroborating information from distances of >100 m [26]. Radar captures regional-scale migration intensity but lacks fine spatial resolution near turbines [10,11,22,51]. Ultrasonic acoustic monitoring detects bats specifically but provides no information about birds. Together, these complementary limitations underscore why the convergence across sensors — rather than any single modality — forms the basis for our taxonomic inference.

Several assumptions underpin the taxonomic inferences drawn from the multi-sensor dataset. First, the strong thermal–radar correlation is interpreted as evidence that birds dominate thermal detections during peak fall migration, which assumes that the regional MTR signal retrieved from KDMX is itself driven primarily by bird migration rather than by bats or insects. This assumption is supported at the seasonal and regional scales NEXRAD samples [10,51] but cannot be verified on a per-night basis. Second, the weak thermal–acoustic correlation is interpreted as evidence that bats contribute modestly to thermal detections during peak migration, but this inference is sensitive to acoustic detectability: ground-mounted microphones sample a smaller volume (~30–50 m) than thermal cameras (out to 200 m), and some migratory bat species suppress echolocation in flight [21] and would be undetectable acoustically even when imaged thermally. The behavioral inference that birds respond to cues associated with blade rotation additionally assumes that rotating and stationary turbines do not differ systematically in ways beyond blade motion that could influence bird behavior; we address that assumption and the broader limits on generalization in the Limitations and Future Directions section below.

### Bird use of rotor-swept altitudes

Across the sampling period, 12,047 3-D flight tracks were documented within 30–150 m above ground level and within 200 m of turbine monopoles, indicating that nocturnal migrants frequently occupy rotor-swept altitudes during inland fall migration. This finding contrasts with the common assumption that most migratory birds travel well above turbine height, though it does not contradict broader evidence that migration often occurs at higher altitudes [31]. Rather, our results suggest that substantial numbers of nocturnal migrants also use lower altitudes, at least under some atmospheric conditions and geographic contexts.

Importantly, birds appeared to dominate detections within this airspace despite well-established evidence that bats experience higher per-megawatt fatality rates than birds at wind energy facilities [1,3,64]. The predominance of birds within rotor-swept altitudes in the airspace next to turbines, coupled with relatively lower mortality rates reported in the literature, supports the hypothesis that behavioral responses play a significant role in reducing collision risk for birds [37,47,65]. The fine-scale trajectory analysis discussed below provides direct evidence bearing on this question.

### Avoidance of the rotor-swept zone by turbine operational status

Fine-scale trajectory analysis revealed that targets approaching turbines under tailwind conditions strongly avoided flight paths directed toward the rotor-swept zone, and that this avoidance was dramatically mediated by turbine operational status even after controlling for wind speed (Fig 5). Targets were 3.6 times more likely to fly toward the RSZ when turbines were stationary than when rotating and producing power, suggesting that operational cues — such as blade motion, aerodynamic noise, or wake turbulence — are primary drivers of the avoidance response rather than the physical structure alone. Avoidance also increased with proximity to turbines, but the distance–avoidance gradient was far steeper when turbines were operating, indicating that birds detect turbine-associated cues at distances of at least 150–200 m and progressively adjust their flight paths as they approach (Fig 5).

Across all four wind direction categories, the majority of targets flew with wind assistance (S1 Fig), consistent with the tailwind-dependent migration strategies of nocturnally migrating birds [66]. The directional gap aligned with the turbine bearing was also evident across wind conditions, suggesting that avoidance of the turbine is not restricted to tailwind scenarios. These patterns are consistent with increasing evidence of avoidance responses by migratory birds near wind turbines [37,39–42,47]. A recent large-scale study [67] combining radar and stereo cameras documented avoidance rates exceeding 99.8% at a near-shore wind farm, with rotor transit rates higher when turbines were idle than when operating. The operational-state dependence we observed parallels those findings and extends them by quantifying the distance-dependent avoidance gradient at individual inland turbines during nocturnal migration. This behavioral capacity may help explain why birds experience lower per-megawatt fatality rates than bats at wind energy facilities [1,3] despite frequently occupying rotor-swept altitudes. However, avoidance behavior is not universal across bird species, and bird fatalities remain of considerable concern, particularly for species with low maneuverability or those attracted to turbine lighting under poor visibility conditions.

The strong operational-state effect also has implications for understanding collision risk more broadly. Curtailment — reducing rotor speed or feathering blades during high-risk periods — is currently implemented primarily to reduce bat fatalities and is not standard practice for migratory songbirds, for which avoidance behavior appears to substantially reduce collision risk. Nonetheless, our finding that stationary turbines elicit far weaker avoidance responses is relevant for interpreting risk during low-wind periods when blades are idle and for informing the design of deterrent systems. If the cues generated by rotating blades are what enable most birds to avoid collisions, then understanding those cues could guide the development of acoustic or visual deterrents that protect species for which natural avoidance is insufficient. Future experimental work — manipulating turbine lighting, sound, or blade rotation speed under matched conditions — would help isolate the specific sensory cues involved.

### Implications for monitoring and mitigation

The strong relationship between regional radar-derived migration intensity and turbine-level thermal detections highlights the practical value of weather radar as a monitoring and risk-assessment tool. Radar data are publicly available, spatially extensive, and require no on-site instrumentation. The correlation between radar activity and local passage rates adjacent to turbines suggests that radar could inform siting decisions by identifying locations or corridors with consistently high nocturnal migration traffic. When paired with localized monitoring methods, radar can also provide broader temporal context for turbine-level activity, helping to characterize the seasonal and nightly variability in bird exposure to turbine airspace.

While curtailment is unlikely to become standard practice for migratory songbirds — given that the avoidance behavior documented here and elsewhere appears to substantially reduce collision risk for most species — our results point toward other practical applications. The strong avoidance response to operating turbines suggests that birds are detecting specific cues associated with blade rotation at distances of over 200 m. Identifying these cues could inform the design of deterrent systems not only for wind turbines but for a broader range of infrastructure where bird collisions are a concern, including communication towers, buildings, and solar facilities. Acoustic or visual deterrents modeled on the stimuli that birds naturally respond to near turbines could be more effective than approaches designed without a mechanistic understanding of avoidance behavior.

A sensor-fusion framework supports progress on both fronts. By combining regional radar with turbine-level thermal and acoustic monitoring, researchers can more confidently infer which taxa are present, when exposure is highest, and how animals behave near structures. Such integrated approaches provide the mechanistic detail needed to develop targeted mitigation strategies while also offering scalable tools for assessing risk across the expanding footprint of wind energy and other infrastructure.

### Limitations and future directions

This study was conducted at two turbines at a single wind facility during fall migration, and the generalizability of these patterns across seasons, regions, and turbine configurations remains unknown. Our thermal cameras were oriented in a single direction, capturing only one approach corridor. While prevailing southward migration during fall makes this the most relevant direction for documenting tailwind-assisted flights, avoidance patterns may differ depending on approach angle relative to turbine orientation and local terrain.

Thermal imaging does not provide species-level identification. Although our multi-sensor comparison provides evidence that birds dominated detections during peak migration, we cannot determine which species were present or whether avoidance varies among taxa. Importantly, the strong avoidance we documented means that the taxonomic composition of targets entering the RSZ may differ substantially from what we observed — species less responsive to turbine cues would be underrepresented in our adjacent-airspace detections but overrepresented among collision victims. Future studies should monitor airspace directly within the RSZ and incorporate both nocturnal flight call recording and bat acoustic monitoring alongside thermal videography and regional radar to enable species-level identification and determine whether avoidance varies taxonomically.

Although our sensitivity analyses indicate the target response to wind turbine operational status effect is robust to wind speed differences, the comparison remains observational, and definitive causal attribution to blade rotation will require experimental manipulation. Future research should examine whether operational-state-dependent avoidance occurs in other regions and seasons. Experimental manipulation of turbine-associated stimuli, including quasi-experimental contrasts using planned curtailment events, would help isolate the sensory mechanisms underlying avoidance and inform the design of deterrent systems applicable to a range of infrastructure where bird collisions are a concern.

## Supporting information

S1 Fig Thermal video ground flight directions filtered by wind direction and wind support. Ground flight directions of thermal video detections binned by 10° intervals, filtered by SCADA wind direction quadrant: north (315°–45°; a), east (45°–135°; b), south (135°–225°; c), and west (225°–315°; d). Bars are colored by wind assistance, defined as whether the animal experienced a tailwind component (tailwind > 0 m/s; orange) or not (teal). Grey shading indicates the direction wind blew towards. The red dashed line at 150° marks the direction toward the wind turbine from space viewed by the thermal cameras.
(TIF)

S1 Text Altitude-capped radar integration vs. thermal correlation. (DOCX)

S2 Text Temporal autocorrelation of hourly detection counts and rationale for nightly aggregation. (DOCX)
