# Supplementary figures and images for: Integrating 3-D thermal videography, ultrasonic acoustics, and weather radar to characterize bird and bat activity at wind turbines
Source: PLoS One. 2026 Jul 14;21(7):e0352329. doi: 10.1371/journal.pone.0352329 (PMC13367684; doi:10.1371/journal.pone.0352329)

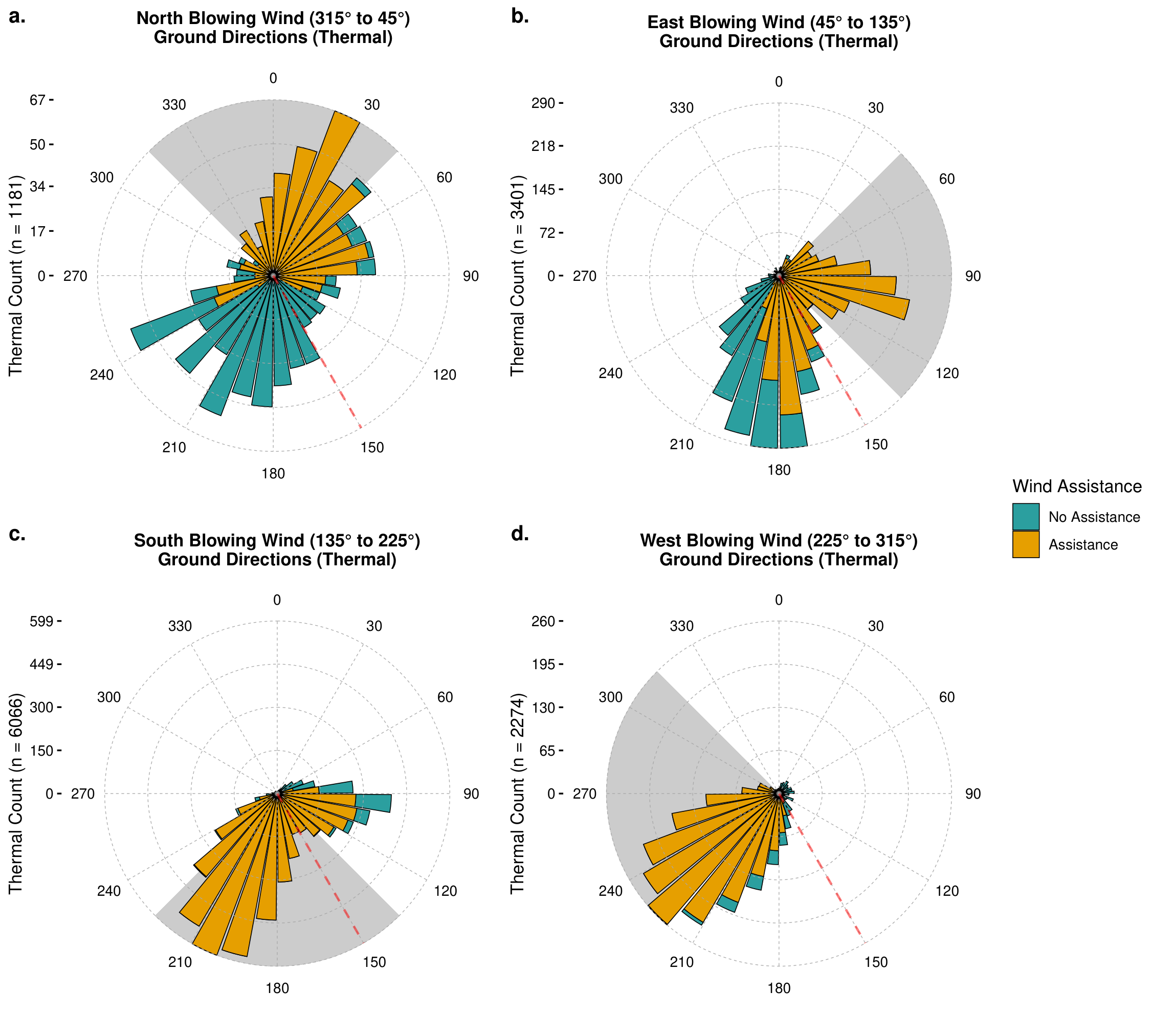

Supplement: S1 Fig — Ground flight directions of thermal video detections binned by 10° intervals, filtered by SCADA wind direction quadrant: north (315°–45°; a), east (45°–135°; b), south (135°–225°; c), and west (225°–315°; d). Bars are colored by wind assistance, defined as whether the animal experienced a tailwind component (tailwind > 0 m/s; orange) or not (teal). Grey shading indicates the direction wind blew towards. The red dashed line at 150° marks the direction toward the wind turbine from space viewed by the thermal cameras. (TIF) [file pone.0352329.s001.tif]
