# Supplementary material for: Integrating 3-D thermal videography, ultrasonic acoustics, and weather radar to characterize bird and bat activity at wind turbines
Source: PLoS One. 2026 Jul 14;21(7):e0352329. doi: 10.1371/journal.pone.0352329 (PMC13367684; doi:10.1371/journal.pone.0352329)
Supplement: S1 Text — (DOCX) [file pone.0352329.s002.docx]

# S1 Text. Altitude-capped radar integration vs. thermal correlation

To address whether restricting KDMX migration traffic rate (MTR) to altitudes closer to the thermal video window (30–150 m above ground level, AGL) would improve its correspondence with turbine-level thermal detections, we re-integrated the bioRad vertical profile time series with increasing altitude caps using integrate_profile (alt_max = 150, 300, 500, 1,000, 2,000 and 5,000 m). Nightly MTR totals were then correlated with nightly thermal video detections summed across the two turbines (n = 40 nights, 2022-08-20 to 2022-10-07).

Bio-Rad’s regularized vertical profile for KDMX is returned on 100 m altitude bins. The lowest three bins (0, 100 and 200 m AGL) contain no valid density estimates in the KDMX product — they are returned as NA in every scan across the study period — likely because of beam-elevation geometry combined with ground-clutter filtering applied during VPR construction. Valid density estimates begin at the 300 m bin (n = 63,123 valid scans; median density 14.5 birds/km³), continue densely up through ~2,800 m, and become sparse above 3,000 m. Consequently, an exact altitude match to the 30–150 m thermal window is not achievable with this product: the lowest altitude cap that includes any valid radar data is alt_max = 500 m, which captures the 300–500 m bins.

At alt_max ≤ 300 m, MTR was uniformly zero (no valid bins within the cap) and correlations were undefined. For alt_max values from 500 to 5,000 m, correlations with nightly thermal totals were essentially unchanged: Pearson r = 0.67–0.68 on raw counts; r = 0.74–0.76 on log-transformed counts; Spearman ρ = 0.73–0.76. Tweedie GLM deviance explained ranged from 0.60 at alt_max = 500 m to 0.63 at alt_max = 5,000 m. Restricting the radar integration to the lowest altitude bins with valid data (alt_max = 500 m) therefore did not strengthen the correspondence, consistent with interpreting the radar signal as a regional integrated index of migration intensity rather than a site-specific measurement of activity over the turbines.
