# Supplementary material for: Integrating 3-D thermal videography, ultrasonic acoustics, and weather radar to characterize bird and bat activity at wind turbines
Source: PLoS One. 2026 Jul 14;21(7):e0352329. doi: 10.1371/journal.pone.0352329 (PMC13367684; doi:10.1371/journal.pone.0352329)
Supplement: S2 Text — (DOCX) [file pone.0352329.s003.docx]

# S2 Text. Temporal autocorrelation of hourly detection counts and rationale for nightly aggregation

All between-sensor relationships reported in the main text were modelled at the nightly scale (sunset to sunrise). Here we document the temporal autocorrelation structure of the underlying hourly data that motivated this choice.

We first paired hourly thermal video detection counts (summed across the two instrumented turbines) with hourly KDMX migration traffic rate values for all hours during the study period in which both sensors had valid data (n = 313 hours across 38 nights). A naive linear model of log(thermal + 1) on log(MTR + 1) produced residuals with a lag-1 autocorrelation of 0.68, decaying slowly over subsequent lags (ACF at lags 1–10: 0.68, 0.48, 0.33, 0.21, 0.14, 0.10, 0.12, 0.09, 0.10, 0.10). A Durbin–Watson statistic of 0.63 and a Ljung–Box test (Q₁₀ = 289, p < 10⁻¹⁶) confirmed strong residual autocorrelation. The same conclusion held when a Tweedie GLM was substituted for the linear model (Ljung–Box Q₁₀ = 263, p < 10⁻¹⁶).

Hourly counts therefore violate the independence assumption required for the Tweedie GLM framework used in the main analyses. Aggregating to nightly totals matches the biologically relevant timescale of nocturnal migration events — which begin shortly after sunset, persist for variable portions of the night, and end at or before sunrise — and produced residuals consistent with white noise (Durbin–Watson 2.26; Ljung–Box Q₁₀ = 7.1, p = 0.71). We therefore used nightly totals in the main Tweedie GLMs.
