# Supplementary material for: Integrating 3-D thermal videography, ultrasonic acoustics, and weather radar to characterize bird and bat activity at wind turbines
Source: PLoS One. 2026 Jul 14;21(7):e0352329. doi: 10.1371/journal.pone.0352329 (PMC13367684; doi:10.1371/journal.pone.0352329)
Supplement: Attachment — Submitted filename: Response_to_Reviewers.docx [file pone.0352329.s005.docx]

**Response to Reviewers**

Manuscript: PONE-D-26-09734

Title: Integrating 3-D Thermal Videography, Ultrasonic Acoustics, and Weather Radar to Characterize Bird and Bat Activity at Wind Turbines

We thank the Academic Editor and both reviewers for their thoughtful and constructive comments. We have responded to every point in detail below and made corresponding revisions to the manuscript. Reviewer and editor comments are reproduced in italics; our responses and the manuscript location of each change follow in regular type.

**Academic Editor: Journal Requirements**

**J1. PLOS ONE style and file-naming requirements**

*Please ensure that your manuscript meets PLOS ONE's style requirements, including those for file naming.*

**Response:** We have reformatted the revised manuscript and supporting information according to the PLOS ONE main-body and title/authors/affiliations templates, and have named all submission files according to the journal's conventions.

**J2. Code sharing for author-generated code**

*PLOS ONE expects all author-generated code to be made available without restrictions upon publication of the work.*

**Response:** All author-generated analysis code and the corresponding de-identified data products are publicly available without restriction at https://github.com/AaronJCorcoran/wind-turbine-multi-sensor-study. Upon acceptance we will archive a versioned release of the repository on Zenodo and cite the resulting DOI. We have added a Data availability section to the revised manuscript describing these arrangements.

**J3. Permits statement**

*In your Methods section, please provide additional information regarding the permits you obtained for the work.*

**Response:** No permits were required because this study was entirely non-invasive and observational: animals were not captured, handled, or disturbed, and monitoring used thermal videography, passive ultrasonic acoustic detectors, and publicly available NEXRAD weather radar. Site access was granted by MidAmerican Energy Company. We have added an explicit Ethics statement to the Methods (Data Collection subsection) stating that no IACUC approval or permit was required, and have acknowledged MidAmerican Energy Company and its technical contributor Jesse Leckband in the Acknowledgements.

**J4. Competing Interests — ThruTracker**

*We note that you received funding from a commercial source: ThruTracker. Please provide an amended Competing Interests Statement that explicitly states this commercial funder, along with any other relevant declarations.*

**Response:** We respectfully clarify a misreading of our original Competing Interests statement. ThruTracker Analytics did not provide any funding for this project. The sole competing interest is that AJC is the developer and owner of ThruTracker Analytics, the commercial software package used to process thermal video data in this study. Funding was provided by the National Renewable Energy Laboratory, not ThruTracker. Our amended Competing Interests statement (included in the cover letter and the revised manuscript) now reads:

*"* *AJC is the owner of ThruTracker Analytics and the developer of ThruTracker, the commercial software package used to process thermal video data in this study. ThruTracker Analytics provided no funding or other support for this work. All other authors declare no competing interests. This does not alter our adherence to PLOS ONE policies on sharing data and materials."*

**J5. Ethics statement**

*Please include your full ethics statement in the 'Methods' section of your manuscript file.*

**Response:** We have added an Ethics statement paragraph to the end of the Data Collection subsection (Methods), which reads: "All data were collected using non-invasive remote-sensing techniques (thermal videography, ultrasonic acoustic detectors, and publicly available weather radar). No animals were captured, handled, or otherwise disturbed during the study, and the work did not involve protected species or sites. Accordingly, no institutional animal care and use committee (IACUC) approval or permit was required. Site access was granted by MidAmerican Energy Company."

**J6. Evaluate suggested citations; J7. Reference list retractions**

**Response:** We reviewed the reference list for retracted citations; none were identified. One new reference was added to support the expanded 3-D videography method description (Theriault et al. 2014, J Exp Biol; now reference [54]).

**Academic Editor: Substantive comments**

**E1. Assumptions and limits paragraph**

*Statements that birds constitute the majority of thermal detections and that avoidance is driven by cues from rotating blades are well supported by patterns in the multi-sensor data, but they still rest on assumptions about radar specificity, acoustic detectability, and potential confounding differences between stationary and operating periods. I suggest adding one short paragraph that explicitly lays out these assumptions and the limits to generalization across sites, seasons, and taxa.*

**Response:** We have added a new paragraph to the Discussion, immediately following the Sensor Concordance and Taxonomic Inference subsection, that explicitly enumerates: (1) the assumption that KDMX MTR is dominated by birds during the study period (citing [10, 51] as seasonal/regional support); (2) the acoustic-detectability issues (smaller ground-detector sampling volume; suppression of echolocation in some migratory bats per [21]); (3) the potential confounds in the stationary–rotating contrast and how our curtailment-based quasi-experimental comparison and wind-speed sensitivity analyses partly address them. The paragraph now appears in the manuscript as the third paragraph of the Discussion (immediately after "This multi-sensor comparison strengthens taxonomic inference in a way that no single monitoring approach could accomplish alone").

**E2. 60–200 m and 30–150 m spatial filter justification**

*The justification for the 60–200 m, 30–150 m spatial filters and the claim that these "effectively exclude insects and airplanes" should either be supported with citations or rephrased a bit more cautiously.*

**Response:** We have softened this claim in the Methods. The revised sentence reads: "These filters also target altitudes and ranges at which insect and aircraft detections are uncommon in our thermal data (A. Corcoran, unpublished data); however, they do not fully exclude such targets, and we retain the possibility that a small number of non-target detections remain in the dataset." An in-preparation study by our group will provide more quantitative support for these filter choices; we did not cite it here because it has not yet reached preprint stage.

**E3. Nightly rather than hourly data for the GLMs**

*Better explanations/justifications of why nightly (rather than hourly) data were used for the GLMs … will help non-specialist readers follow your modeling choices.*

**Response:** We re-ran the hourly analysis in response to this comment and found that hourly counts within nights are strongly temporally autocorrelated (Ljung–Box test for residual autocorrelation in a naive hourly log–log model: Q₁₀ = 289, p < 10⁻¹⁶; lag-1 autocorrelation = 0.68; Durbin–Watson = 0.63), violating the independence assumption underlying the Tweedie GLM framework used here. Aggregating to nightly totals matches the biologically relevant timescale of nocturnal migration events and yields residuals consistent with white noise (Durbin–Watson = 2.26; Ljung–Box Q₁₀ = 7.1, p = 0.71). The revised Methods explains this rationale concisely and the new S2 Appendix of the Supporting Information reports the full autocorrelation diagnostics. We chose not to report modelled p-values from AR(1)-corrected hourly GLS fits because those fits still rest on the same underlying data whose independence assumption is violated at the hourly scale.

**E4. Turbine ID as a fixed effect in the mixed model**

*…why turbine ID is treated as a fixed effect in the mixed model will help non-specialist readers follow your modeling choices.*

**Response:** We have expanded the justification in the Methods (Statistical Analysis subsection) to state that turbine identity was treated as a fixed effect rather than a random effect for two reasons: (i) random-effect variance components require multiple grouping levels (typically ≥ 5–6) to be estimated reliably, and (ii) the two turbines represent the full set of turbines monitored at this site rather than a random sample from a broader turbine population, making fixed-effect treatment inferentially appropriate. Night continues to be included as a random intercept.

**E5. Figure resolution and legibility**

**Response:** All main-text figures (Figs 2–5) and supplemental Figure S1 have been re-exported at 600 dpi from the original ggplot2 source (PNG and PDF versions both provided). Figure-specific changes are detailed in the responses to Reviewer 2 below.

**Reviewer #1**

**R1-1. Figure resolution**

*The figures appear blurry and low resolution. Please provide higher-resolution versions to improve readability.*

**Response:** All main-text and supplemental figures have been re-exported at 600 dpi and, in the process, reviewed for legibility (tick label size, contrasting colors for categorical variables, and explicit legend items for shaded regions). Both PNG and PDF versions are provided.

**Reviewer #2**

**R2-1. Distance from KDMX to the sampling site**

*How far from the KDMX radar were the sampling sites? I ask since the vertical profiles from bioRad are constructed from data within 35 km of the radar. The farther from the radar the sampling sites are the lower the accuracy of the radar measures may be relative to activity at the sampling site, particularly if the sampling site is beyond 35 km from the radar.*

**Response:** We measured the distance from KDMX (41.7311 °N, –93.7228 °W) to the Orient Wind Farm (41.198 °N, –94.417 °W) as approximately 83 km, well beyond the 35 km window within which bioRad's low-altitude VPR retrievals are most reliable. We have added this distance and a brief discussion of its implications to the Methods: the radar data are used as a regional index of migration intensity integrated across the broader radar-coverage region, rather than as a site-specific measurement directly above the turbines. This framing is also reflected in the revised Discussion and limitations paragraph.

**R2-2. Altitude-matched radar-thermal comparison**

*It seems radar based migration traffic rate is considering birds flying up to 5 km above ground. But thermal data are restricted to targets flying between 30 and 150 m above ground. … could you also compare the radar traffic to thermal traffic by restricting the radar data to within 150 m above ground to make it more of an apples to apples comparison? Would that improve the correlation even more?*

**Response:** We re-ran the bioRad integration at a range of altitude caps (alt_max = 150, 300, 500, 1,000, 2,000 and 5,000 m) and correlated the resulting nightly MTR totals with nightly thermal detections. Two points are worth stating explicitly in light of the reviewer's comment. First, bioRad does integrate across the full ~5–35 km range ring around the radar, so the vertical profile in principle receives contributions from low altitudes at close range. However, the KDMX VPR product does not return density estimates below 300 m AGL: the 0, 100 and 200 m altitude bins are returned as NA in every scan (likely as a consequence of beam-elevation geometry combined with ground-clutter filtering applied during VPR construction), and valid density estimates begin at the 300 m bin. An exact altitude match to the 30–150 m thermal window is therefore not achievable with this product. Second, the lowest cap that does include valid radar data (alt_max = 500 m, which captures the 300–500 m bins) produced a correlation with nightly thermal detections (Pearson r ≈ 0.67) that was not improved by raising the cap further (r = 0.67–0.68 on raw counts and r = 0.74–0.76 on log-transformed counts across alt_max = 500–5,000 m; Tweedie GLM deviance explained 0.60–0.63). Restricting to the lowest altitudes with valid radar data did not strengthen the correspondence, consistent with interpreting the radar signal as a regional integrated index of migration intensity rather than a site-specific measurement directly above the turbines. We have added this clarification to the Methods and report the full altitude-cap comparison in the new S1 Appendix.

**R2-3. Details of 3-D detection generation**

*Please provide more detail as to how your algorithms generate 3-D detections from thermal video information. There is broad variability in bird size and flight speed, so those may not be reliable indicators of altitude.*

**Response:** We have expanded the Data Processing paragraph in Methods to describe the pipeline more fully: paired, frame-synchronized Flir A65 cameras at each turbine feed a YOLOv3-based 2-D detector per camera view; intrinsic and extrinsic calibration data support a direct linear transformation (DLT; Theriault et al. 2014 [54]) that reconstructs real-world 3-D positions by triangulating matched detections across the two overlapping views. The revision explicitly states: "Altitude and three-dimensional position therefore derive from stereo parallax and camera geometry alone; target size and flight speed are not used as altitude proxies, so inter-individual variation in those traits does not bias altitude estimates." The reprojection-residual-based error statistics previously reported remain unchanged.

**R2-4. Figure 3 — axis labels, scientific notation, shading**

*Please do not use scientific notation for the values on the x-axis for panel a. It is difficult to interpret them as they run together. Also, because the axes use logarithmic scaling for raw values, the labels should reflect the original variable name rather than 'log [Variable].' Only label it as 'log' if the numerical values shown on the scale have already been transformed. What do the shaded regions around trendlines represent?*

**Response:** We have revised Figure 3 as follows: (i) replaced the scientific-notation tick labels with comma-separated plain numerals; (ii) removed "log" from axis titles, since the displayed tick values are on the original (untransformed) scale — the axes use log10 scaling only for layout; (iii) updated the caption to explicitly state that solid lines are GLM fitted values and shaded bands are 95% confidence intervals on the fitted mean.

**R2-5. Figure 5 — two trendlines; shading**

*Why are there two trendlines for each type of wind activity (rotating or stationary)? What do the shaded regions around trendlines represent?*

**Response:** Thank you for flagging this. The two GAM trendlines per status (rotating vs stationary) are deliberate and reflect the underlying bimodal flight-direction distribution: as targets approach the turbine they deviate either above or below the 150° turbine bearing (i.e., to either side of the turbine), and we fit separate smooths to tracks with camera-frame flight directions ≥ 150° and < 150° to visualize both branches of the avoidance response. We updated the Figure 5 caption to explain this explicitly and to state that the shaded bands around each smooth are 95% confidence intervals on the fitted mean.

We thank the Editor and reviewers again for their careful reading and constructive suggestions, which we believe have materially improved the manuscript. We hope that the revisions address all concerns and look forward to your response.
