# Supplementary material for: Integrating 3-D thermal videography, ultrasonic acoustics, and weather radar to characterize bird and bat activity at wind turbines
Source: PLoS One. 2026 Jul 14;21(7):e0352329. doi: 10.1371/journal.pone.0352329 (PMC13367684; doi:10.1371/journal.pone.0352329)
Supplement: Peer review history

# Peer review: Decision Letter 0

**DOI:** 10.1371/journal.pone.0352329.r001

**Authors:** James Sheppard

20 Apr 2026

PONE-D-26-09734

Integrating 3-D Thermal Videography, Ultrasonic Acoustics, and Weather Radar to Characterize Bird and Bat Activity at Wind Turbines

PLOS One

Dear Dr. Corcoran,

Thank you for submitting your manuscript to PLOS ONE. After careful consideration, we feel that it has merit but does not fully meet PLOS ONE’s publication criteria as it currently stands. Therefore, we invite you to submit a revised version of the manuscript that addresses the points raised during the review process.

Please submit your revised manuscript by Jun 04 2026 11:59PM. If you will need more time than this to complete your revisions, please reply to this message or contact the journal office at plosone@plos.org. When you're ready to submit your revision, log on to https://www.editorialmanager.com/pone/ and select the ’submissions Needing Revision' folder to locate your manuscript file.

Please include the following items when submitting your revised manuscript:

- A letter that responds to each point raised by the academic editor and reviewer(s). You should upload this letter as a separate file labeled 'Response to Reviewers'.
- A marked-up copy of your manuscript that highlights changes made to the original version. You should upload this as a separate file labeled 'Revised Manuscript with Track Changes'.
- An unmarked version of your revised paper without tracked changes. You should upload this as a separate file labeled 'Manuscript'.

If you would like to make changes to your financial disclosure, please include your updated statement in your cover letter. Guidelines for resubmitting your figure files are available below the reviewer comments at the end of this letter.

If applicable, we recommend that you deposit your laboratory protocols in protocols.io to enhance the reproducibility of your results. Protocols.io assigns your protocol its own identifier (DOI) so that it can be cited independently in the future. For instructions see: https://journals.plos.org/plosone/s/submission-guidelines#loc-laboratory-protocols. Additionally, PLOS ONE offers an option for publishing peer-reviewed Lab Protocol articles, which describe protocols hosted on protocols.io. Read more information on sharing protocols at https://plos.org/protocols?utm_medium=editorial-email&utm_source=authorletters&utm_campaign=protocols.

As the corresponding author, your ORCID iD is verified in the submission system and will appear in the published article. PLOS supports the use of ORCID, and we encourage all coauthors to register for an ORCID iD and use it as well. Please encourage your coauthors to verify their ORCID iD within the submission system before final acceptance, as unverified ORCID iDs will not appear in the published article. *Only*  the individual author can complete the verification step; PLOS staff *cannot*  verify ORCID iDs on behalf of authors.

We look forward to receiving your revised manuscript.

Kind regards,

James K. Sheppard

Academic Editor

PLOS One

Journal Requirements:

When submitting your revision, we need you to address these additional requirements.

1.Please ensure that your manuscript meets PLOS ONE’s style requirements, including those for file naming. The PLOS ONE style templates can be found at

https://journals.plos.org/plosone/s/file?id=wjVg/PLOSOne_formatting_sample_main_body.pdf and

https://journals.plos.org/plosone/s/file?id=ba62/PLOSOne_formatting_sample_title_authors_affiliations.pdf

2. Please note that PLOS One has specific guidelines on code sharing for submissions in which author-generated code underpins the findings in the manuscript. In these cases, we expect all author-generated code to be made available without restrictions upon publication of the work. Please review our guidelines at https://journals.plos.org/plosone/s/materials-and-software-sharing#loc-sharing-code and ensure that your code is shared in a way that follows best practice and facilitates reproducibility and reuse.

3. In your Methods section, please provide additional information regarding the permits you obtained for the work. Please ensure you have included the full name of the authority that approved the field site access and, if no permits were required, a brief statement explaining why.

4. Thank you for stating the following in the Competing Interests section:

“We have read the journal’s policy and ajc has a competing interest of being the developer of ThruTracker, a commercially available software package used in this study. All other authors declare no competing interests.”

We note that you received funding from a commercial source: “ThruTracker”

Please provide an amended Competing Interests Statement that explicitly states this commercial funder, along with any other relevant declarations relating to employment, consultancy, patents, products in development, marketed products, etc.

Within this Competing Interests Statement, please confirm that this does not alter your adherence to all PLOS ONE policies on sharing data and materials by including the following statement: "This does not alter our adherence to PLOS ONE policies on sharing data and materials.” (as detailed online in our guide for authors http://journals.plos.org/plosone/s/competing-interests).  If there are restrictions on sharing of data and/or materials, please state these. Please note that we cannot proceed with consideration of your article until this information has been declared.

Please include your amended Competing Interests Statement within your cover letter. We will change the online submission form on your behalf.

5. Please include your full ethics statement in the ‘Methods’ section of your manuscript file. In your statement, please include the full name of the IRB or ethics committee who approved or waived your study, as well as whether or not you obtained informed written or verbal consent. If consent was waived for your study, please include this information in your statement as well.

6. If the reviewer comments include a recommendation to cite specific previously published works, please review and evaluate these publications to determine whether they are relevant and should be cited. There is no requirement to cite these works unless the editor has indicated otherwise.

7. Please review your reference list to ensure that it is complete and correct. If you have cited papers that have been retracted, please include the rationale for doing so in the manuscript text, or remove these references and replace them with relevant current references. Any changes to the reference list should be mentioned in the rebuttal letter that accompanies your revised manuscript. If you need to cite a retracted article, indicate the article’s retracted status in the References list and also include a citation and full reference for the retraction notice.

Additional Editor Comments:

This is a strong, technically sound manuscript, and the remaining issues are pretty minor clarifications and presentation issues. Statements that birds constitute the majority of thermal detections and that avoidance is driven by cues from rotating blades are well supported by patterns in the multi-sensor data, but they still rest on assumptions about radar specificity, acoustic detectability, and potential confounding differences between stationary and operating periods. I suggest adding one short paragraph that explicitly lays out these assumptions and the limits to generalization across sites, seasons, and taxa (echoing the reviewers’ requests for clearer discussion of uncertainty).

The justification for the 60–200 m, 30–150 m spatial filters and the claim that these “effectively exclude insects and airplanes” should either be supported with citations or rephrased a bit more cautiously. Better explanations/justifications of why nightly (rather than hourly) data were used for the GLMs, and why turbine ID is treated as a fixed effect in the mixed model, will help non-specialist readers follow your modeling choices. Both reviewers note that figure resolution and legibility need improvement, so providing higher-resolution figures with fully explained symbols, colors etc will improve manuscript clarity. Consequently, minor changes are required before the manuscript can be further considered. Both reviewers provide insightful comments and suggestions that will help guide the revision process to improve this manuscript.

[Note: HTML markup is below. Please do not edit.]

Reviewer’s Responses to Questions

**Comments to the Author**

1. Is the manuscript technically sound, and do the data support the conclusions?

The manuscript must describe a technically sound piece of scientific research with data that supports the conclusions. Experiments must have been conducted rigorously, with appropriate controls, replication, and sample sizes. The conclusions must be drawn appropriately based on the data presented.

Reviewer #1: Yes

Reviewer #2: Yes

**********

2. Has the statistical analysis been performed appropriately and rigorously?

Reviewer #1: Yes

Reviewer #2: Yes

**********

3. Have the authors made all data underlying the findings in their manuscript fully available?

The PLOS Data policy requires authors to make all data underlying the findings described in their manuscript fully available without restriction, with rare exception (please refer to the Data Availability Statement in the manuscript PDF file). The data should be provided as part of the manuscript or its supporting information, or deposited to a public repository. For example, in addition to summary statistics, the data points behind means, medians and variance measures should be available. If there are restrictions on publicly sharing data—e.g. participant privacy or use of data from a third party—those must be specified.

Reviewer #1: Yes

Reviewer #2: Yes

**********

4. Is the manuscript presented in an intelligible fashion and written in standard English?

PLOS ONE does not copyedit accepted manuscripts, so the language in submitted articles must be clear, correct, and unambiguous. Any typographical or grammatical errors should be corrected at revision, so please note any specific errors here.

Reviewer #1: Yes

Reviewer #2: Yes

**********

5. Review Comments to the Author

Please use the space provided to explain your answers to the questions above. You may also include additional comments for the author, including concerns about dual publication, research ethics, or publication ethics. (Please upload your review as an attachment if it exceeds 20,000 characters)

Reviewer #1: The manuscript is generally well structured, provides detailed descriptions of the methodology, such as data collection and data processing procedures, and includes rigorous statistical analyses supporting the findings. The figures appear blurry and low resolution. Please provide higher-resolution versions to improve readability.

Reviewer #2: This manuscript is clearly written and describes a sound study design and appropriate statistical analyses. The interpretation of the results are reasonable and don't stray into the realm of speculation. I only have a few minor comments to improve presentation of data in figures and to clarify some aspects of the radar data and analysis.

Line 128 How far from the KDMX radar were the sampling sites? I ask since the vertical profiles from bioRad are constructed from data within 35 km of the radar. The farther from the radar the sampling sites are the lower the accuracy of the radar measures may be relative to activity at the sampling site, particularly if the sampling site is beyond 35 km from the radar.

Line 132 It seems radar based migration traffic rate is considering birds fliying up to 5 km above ground. But thermal data are restricted to targets flying between 30 and 150 m above ground. The correlation between the two measures is rather tight already, but could you also compare the radar traffic to thermal traffic by restricting the radar data to within 150 m above ground to make it more of an apples to apples comparison? Would that improve the correlation even more?

Line 145. Please provide more detail as to how your algorithms generate 3-D detections from thermal video information. There is broad variability in bird size and flight speed, so thsoe may not be reliable indicators of altitude. I recall

Figure 3. Please do not use scientific notation for the values on the x-axis for panel a. It is difficult to interpret them as them run together. Also, because the axes use logarithmic scaling for raw values, the labels should reflect the original variable name rather than 'log [Variable].' Only label it as 'log' if the numerical values shown on the scale have already been transformed. What do the shaded regions around trendlines represent?

Figure 5. Why are there two trendlines for each type of wind activity (rotating or stationary)? What do the shaded regions around trendlines represent?

**********

6. PLOS authors have the option to publish the peer review history of their article (what does this mean?). If published, this will include your full peer review and any attached files.

If you choose “no”, your identity will remain anonymous but your review may still be made public.

**Do you want your identity to be public for this peer review?**  For information about this choice, including consent withdrawal, please see our Privacy Policy.

Reviewer #1: No

Reviewer #2: No

**********

[NOTE: If reviewer comments were submitted as an attachment file, they will be attached to this email and accessible via the submission site. Please log into your account, locate the manuscript record, and check for the action link "View Attachments". If this link does not appear, there are no attachment files.]

To ensure your figures meet our technical requirements, please review our figure guidelines: https://journals.plos.org/plosone/s/figures

You may also use PLOS’s free figure tool, NAAS, to help you prepare publication quality figures: https://journals.plos.org/plosone/s/figures#loc-tools-for-figure-preparation.

NAAS will assess whether your figures meet our technical requirements by comparing each figure against our figure specifications.

# Author comment: Author response to Decision Letter 1

**DOI:** 10.1371/journal.pone.0352329.r002

13 May 2026

Response to Reviewers

Manuscript: PONE-D-26-09734

Title: Integrating 3-D Thermal Videography, Ultrasonic Acoustics, and Weather Radar to Characterize Bird and Bat Activity at Wind Turbines

We thank the Academic Editor and both reviewers for their thoughtful and constructive comments. We have responded to every point in detail below and made corresponding revisions to the manuscript. Reviewer and editor comments are reproduced in italics; our responses and the manuscript location of each change follow in regular type.

Academic Editor: Journal Requirements

J1. PLOS ONE style and file-naming requirements

Please ensure that your manuscript meets PLOS ONE’s style requirements, including those for file naming.

Response: We have reformatted the revised manuscript and supporting information according to the PLOS ONE main-body and title/authors/affiliations templates, and have named all submission files according to the journal’s conventions.

J2. Code sharing for author-generated code

PLOS ONE expects all author-generated code to be made available without restrictions upon publication of the work.

Response: All author-generated analysis code and the corresponding de-identified data products are publicly available without restriction at https://github.com/AaronJCorcoran/wind-turbine-multi-sensor-study. Upon acceptance we will archive a versioned release of the repository on Zenodo and cite the resulting DOI. We have added a Data availability section to the revised manuscript describing these arrangements.

J3. Permits statement

In your Methods section, please provide additional information regarding the permits you obtained for the work.

Response: No permits were required because this study was entirely non-invasive and observational: animals were not captured, handled, or disturbed, and monitoring used thermal videography, passive ultrasonic acoustic detectors, and publicly available NEXRAD weather radar. Site access was granted by MidAmerican Energy Company. We have added an explicit Ethics statement to the Methods (Data Collection subsection) stating that no IACUC approval or permit was required, and have acknowledged MidAmerican Energy Company and its technical contributor Jesse Leckband in the Acknowledgements.

J4. Competing Interests — ThruTracker

We note that you received funding from a commercial source: ThruTracker. Please provide an amended Competing Interests Statement that explicitly states this commercial funder, along with any other relevant declarations.

Response: We respectfully clarify a misreading of our original Competing Interests statement. ThruTracker Analytics did not provide any funding for this project. The sole competing interest is that AJC is the developer and owner of ThruTracker Analytics, the commercial software package used to process thermal video data in this study. Funding was provided by the National Renewable Energy Laboratory, not ThruTracker. Our amended Competing Interests statement (included in the cover letter and the revised manuscript) now reads:

" AJC is the owner of ThruTracker Analytics and the developer of ThruTracker, the commercial software package used to process thermal video data in this study. ThruTracker Analytics provided no funding or other support for this work. All other authors declare no competing interests. This does not alter our adherence to PLOS ONE policies on sharing data and materials."

J5. Ethics statement

Please include your full ethics statement in the 'Methods' section of your manuscript file.

Response: We have added an Ethics statement paragraph to the end of the Data Collection subsection (Methods), which reads: "All data were collected using non-invasive remote-sensing techniques (thermal videography, ultrasonic acoustic detectors, and publicly available weather radar). No animals were captured, handled, or otherwise disturbed during the study, and the work did not involve protected species or sites. Accordingly, no institutional animal care and use committee (IACUC) approval or permit was required. Site access was granted by MidAmerican Energy Company."

J6. Evaluate suggested citations; J7. Reference list retractions

Response: We reviewed the reference list for retracted citations; none were identified. One new reference was added to support the expanded 3-D videography method description (Theriault et al. 2014, J Exp Biol; now reference [54]).

Academic Editor: Substantive comments

E1. Assumptions and limits paragraph

Statements that birds constitute the majority of thermal detections and that avoidance is driven by cues from rotating blades are well supported by patterns in the multi-sensor data, but they still rest on assumptions about radar specificity, acoustic detectability, and potential confounding differences between stationary and operating periods. I suggest adding one short paragraph that explicitly lays out these assumptions and the limits to generalization across sites, seasons, and taxa.

Response: We have added a new paragraph to the Discussion, immediately following the Sensor Concordance and Taxonomic Inference subsection, that explicitly enumerates: (1) the assumption that KDMX MTR is dominated by birds during the study period (citing [10, 51] as seasonal/regional support); (2) the acoustic-detectability issues (smaller ground-detector sampling volume; suppression of echolocation in some migratory bats per [21]); (3) the potential confounds in the stationary–rotating contrast and how our curtailment-based quasi-experimental comparison and wind-speed sensitivity analyses partly address them. The paragraph now appears in the manuscript as the third paragraph of the Discussion (immediately after "This multi-sensor comparison strengthens taxonomic inference in a way that no single monitoring approach could accomplish alone").

E2. 60–200 m and 30–150 m spatial filter justification

The justification for the 60–200 m, 30–150 m spatial filters and the claim that these "effectively exclude insects and airplanes" should either be supported with citations or rephrased a bit more cautiously.

Response: We have softened this claim in the Methods. The revised sentence reads: "These filters also target altitudes and ranges at which insect and aircraft detections are uncommon in our thermal data (A. Corcoran, unpublished data); however, they do not fully exclude such targets, and we retain the possibility that a small number of non-target detections remain in the dataset." An in-preparation study by our group will provide more quantitative support for these filter choices; we did not cite it here because it has not yet reached preprint stage.

E3. Nightly rather than hourly data for the GLMs

Better explanations/justifications of why nightly (rather than hourly) data were used for the GLMs … will help non-specialist readers follow your modeling choices.

Response: We re-ran the hourly analysis in response to this comment and found that hourly counts within nights are strongly temporally autocorrelated (Ljung–Box test for residual autocorrelation in a naive hourly log–log model: Q₁₀ = 289, p < 10⁻¹⁶; lag-1 autocorrelation = 0.68; Durbin–Watson = 0.63), violating the independence assumption underlying the Tweedie GLM framework used here. Aggregating to nightly totals matches the biologically relevant timescale of nocturnal migration events and yields residuals consistent with white noise (Durbin–Watson = 2.26; Ljung–Box Q₁₀ = 7.1, p = 0.71). The revised Methods explains this rationale concisely and the new S2 Appendix of the Supporting Information reports the full autocorrelation diagnostics. We chose not to report modelled p-values from AR(1)-corrected hourly GLS fits because those fits still rest on the same underlying data whose independence assumption is violated at the hourly scale.

E4. Turbine ID as a fixed effect in the mixed model

…why turbine ID is treated as a fixed effect in the mixed model will help non-specialist readers follow your modeling choices.

Response: We have expanded the justification in the Methods (Statistical Analysis subsection) to state that turbine identity was treated as a fixed effect rather than a random effect for two reasons: (i) random-effect variance components require multiple grouping levels (typically ≥ 5–6) to be estimated reliably, and (ii) the two turbines represent the full set of turbines monitored at this site rather than a random sample from a broader turbine population, making fixed-effect treatment inferentially appropriate. Night continues to be included as a random intercept.

E5. Figure resolution and legibility

Response: All main-text figures (Figs 2–5) and supplemental Figure S1 have been re-exported at 600 dpi from the original ggplot2 source (PNG and PDF versions both provided). Figure-specific changes are detailed in the responses to Reviewer 2 below.

Reviewer #1

R1-1. Figure resolution

The figures appear blurry and low resolution. Please provide higher-resolution versions to improve readability.

Response: All main-text and supplemental figures have been re-exported at 600 dpi and, in the process, reviewed for legibility (tick label size, contrasting colors for categorical variables, and explicit legend items for shaded regions). Both PNG and PDF versions are provided.

Reviewer #2

R2-1. Distance from KDMX to the sampling site

How far from the KDMX radar were the sampling sites? I ask since the vertical profiles from bioRad are constructed from data within 35 km of the radar. The farther from the radar the sampling sites are the lower the accuracy of the radar measures may be relative to activity at the sampling site, particularly if the sampling site is beyond 35 km from the radar.

Response: We measured the distance from KDMX (41.7311 °N, –93.7228 °W) to the Orient Wind Farm (41.198 °N, –94.417 °W) as approximately 83 km, well beyond the 35 km window within which bioRad’s low-altitude VPR retrievals are most reliable. We have added this distance and a brief discussion of its implications to the Methods: the radar data are used as a regional index of migration intensity integrated across the broader radar-coverage region, rather than as a site-specific measurement directly above the turbines. This framing is also reflected in the revised Discussion and limitations paragraph.

R2-2. Altitude-matched radar-thermal comparison

It seems radar based migration traffic rate is considering birds flying up to 5 km above ground. But thermal data are restricted to targets flying between 30 and 150 m above ground. … could you also compare the radar traffic to thermal traffic by restricting the radar data to within 150 m above ground to make it more of an apples to apples comparison? Would that improve the correlation even more?

Response: We re-ran the bioRad integration at a range of altitude caps (alt_max = 150, 300, 500, 1,000, 2,000 and 5,000 m) and correlated the resulting nightly MTR totals with nightly thermal detections. Two points are worth stating explicitly in light of the reviewer’s comment. First, bioRad does integrate across the full ~5–35 km range ring around the radar, so the vertical profile in principle receives contributions from low altitudes at close range. However, the KDMX VPR product does not return density estimates below 300 m AGL: the 0, 100 and 200 m altitude bins are returned as NA in every scan (likely as a consequence of beam-elevation geometry combined with ground-clutter filtering applied during VPR construction), and valid density estimates begin at the 300 m bin. An exact altitude match to the 30–150 m thermal window is therefore not achievable with this product. Second, the lowest cap that does include valid radar data (alt_max = 500 m, which captures the 300–500 m bins) produced a correlation with nightly thermal detections (Pearson r ≈ 0.67) that was not improved by raising the cap further (r = 0.67–0.68 on raw counts and r = 0.74–0.76 on log-transformed counts across alt_max = 500–5,000 m; Tweedie GLM deviance explained 0.60–0.63). Restricting to the lowest altitudes with valid radar data did not strengthen the correspondence, consistent with interpreting the radar signal as a regional integrated index of migration intensity rather than a site-specific measurement directly above the turbines. We have added this clarification to the Methods and report the full altitude-cap comparison in the new S1 Appendix.

R2-3. Details of 3-D detection generation

Please provide more detail as to how your algorithms generate 3-D detections from thermal video information. There is broad variability in bird size and flight speed, so those may not be reliable indicators of altitude.

Response: We have expanded the Data Processing paragraph in Methods to describe the pipeline more fully: paired, frame-synchronized Flir A65 cameras at each turbine feed a YOLOv3-based 2-D detector per camera view; intrinsic and extrinsic calibration data support a direct linear transformation (DLT; Theriault et al. 2014 [54]) that reconstructs real-world 3-D positions by triangulating matched detections across the two overlapping views. The revision explicitly states: "Altitude and three-dimensional position therefore derive from stereo parallax and camera geometry alone; target size and flight speed are not used as altitude proxies, so inter-individual variation in those traits does not bias altitude estimates." The reprojection-residual-based error statistics previously reported remain unchanged.

R2-4. Figure 3 — axis labels, scientific notation, shading

Please do not use scientific notation for the values on the x-axis for panel a. It is difficult to interpret them as they run together. Also, because the axes use logarithmic scaling for raw values, the labels should reflect the original variable name rather than 'log [Variable].' Only label it as 'log' if the numerical values shown on the scale have already been transformed. What do the shaded regions around trendlines represent?

Response: We have revised Figure 3 as follows: (i) replaced the scientific-notation tick labels with comma-separated plain numerals; (ii) removed "log" from axis titles, since the displayed tick values are on the original (untransformed) scale — the axes use log10 scaling only for layout; (iii) updated the caption to explicitly state that solid lines are GLM fitted values and shaded bands are 95% confidence intervals on the fitted mean.

R2-5. Figure 5 — two trendlines; shading

Why are there two trendlines for each type of wind activity (rotating or stationary)? What do the shaded regions around trendlines represent?

Response: Thank you for flagging this. The two GAM trendlines per status (rotating vs stationary) are deliberate and reflect the underlying bimodal flight-direction distribution: as targets approach the turbine they deviate either above or below the 150° turbine bearing (i.e., to either side of the turbine), and we fit separate smooths to tracks with camera-frame flight directions ≥ 150° and < 150° to visualize both branches of the avoidance response. We updated the Figure 5 caption to explain this explicitly and to state that the shaded bands around each smooth are 95% confidence intervals on the fitted mean.

We thank the Editor and reviewers again for their careful reading and constructive suggestions, which we believe have materially improved the manuscript. We hope that the revisions address all concerns and look forward to your response.

Attachment Submitted filename: Response_to_Reviewers.docx

# Editor report: Decision Letter 1

**DOI:** 10.1371/journal.pone.0352329.r003

**Authors:** James Sheppard, James Sheppard

10 Jun 2026

<p>Integrating 3-D Thermal Videography, Ultrasonic Acoustics, and Weather Radar to Characterize Bird and Bat Activity at Wind Turbines

PONE-D-26-09734R1

Dear Dr. Corcoran,

We’re pleased to inform you that your manuscript has been judged scientifically suitable for publication and will be formally accepted for publication once it meets all outstanding technical requirements.

Within one week, you’ll receive an e-mail detailing the required amendments. When these have been addressed, you’ll receive a formal acceptance letter and your manuscript will be scheduled for publication.

An invoice will be generated when your article is formally accepted. Please note, if your institution has a publishing partnership with PLOS and your article meets the relevant criteria, all or part of your publication costs will be covered. Please make sure your user information is up-to-date by logging into Editorial Manager at Editorial Manager® and clicking the ‘Update My Information' link at the top of the page. For questions related to billing, please contact billing support.

If your institution or institutions have a press office, please notify them about your upcoming paper to help maximize its impact. If they’ll be preparing press materials, please inform our press team as soon as possible -- no later than 48 hours after receiving the formal acceptance. Your manuscript will remain under strict press embargo until 2 pm Eastern Time on the date of publication. For more information, please contact onepress@plos.org.

Kind regards,

James K. Sheppard

Academic Editor

PLOS One

Additional Editor Comments (optional):

The authors have satisfactorily addressed the minor concerns raised by both reviewers and myself. They have clarified the key assumptions underlying their taxonomic and behavioral inferences, provided additional justification for their spatial and analytical choices, expanded the description of the 3‑D detection methods, and supplied the requested radar comparisons. Figure quality and labeling have been improved, and the ethics, data, and competing‑interests statements now meet requirements. In my judgment, the manuscript is now suitable for publication in PLOS One and will be of clear relevance and value to wildlife professionals working at the interface of ecology and wind‑energy development.

Reviewers' comments:

# Editor report: Acceptance letter

**DOI:** 10.1371/journal.pone.0352329.r004

**Authors:** James Sheppard, James Sheppard

PONE-D-26-09734R1

PLOS One

Dear Dr. Corcoran,

I'm pleased to inform you that your manuscript has been deemed suitable for publication in PLOS One. Congratulations! Your manuscript is now being handed over to our production team.

At this stage, our production department will prepare your paper for publication. This includes ensuring the following:

* All references, tables, and figures are properly cited

* All relevant supporting information is included in the manuscript submission,

* There are no issues that prevent the paper from being properly typeset

You will receive further instructions from the production team, including instructions on how to review your proof when it is ready. Please keep in mind that we are working through a large volume of accepted articles, so please give us a few days to review your paper and let you know the next and final steps.

Lastly, if your institution or institutions have a press office, please let them know about your upcoming paper now to help maximize its impact. If they'll be preparing press materials, please inform our press team within the next 48 hours. Your manuscript will remain under strict press embargo until 2 pm Eastern Time on the date of publication. For more information, please contact onepress@plos.org.

You will receive an invoice from PLOS for your publication fee after your manuscript has reached the completed accept phase. If you receive an email requesting payment before acceptance or for any other service, this may be a phishing scheme. Learn how to identify phishing emails and protect your accounts at https://explore.plos.org/phishing.

If we can help with anything else, please email us at customercare@plos.org.

Thank you for submitting your work to PLOS ONE and supporting open access.

Kind regards,

PLOS ONE Editorial Office Staff

on behalf of

Dr. James K. Sheppard

Academic Editor

PLOS One
